# Unhealthy alcohol use and intimate partner violence among men and women living with HIV in Uganda

**DOI:** 10.1186/s12889-022-14295-2

**Published:** 2022-10-10

**Authors:** Amanda P. Miller, Robin Fatch, Sara Lodi, Kara Marson, Nneka Emenyonu, Allen Kekibiina, Brian Beesiga, Gabriel Chamie, Winnie R. Muyindike, Judith A. Hahn

**Affiliations:** 1 Division of Infectious Diseases, David Geffen School of Medicine, University of California, Los Angeles, CA USA; 2 Division of HIV, Infectious Diseases, and Global Medicine, Department of Medicine, University of California San Francisco, San Francisco, CA USA; 3 Boston University School of Public Health, Boston, MA USA; 4 Global Health Collaborative, Mbarara University of Science and Technology, Mbarara, Uganda; 5 Infectious Diseases Research Collaboration, Kampala, Uganda; 6 Mbarara Regional Referral Hospital, Mbarara, Uganda

**Keywords:** Intimate partner violence, Alcohol use, HIV, Uganda

## Abstract

**Background:**

Intimate partner violence (IPV) and alcohol use are interrelated public health issues. Heavy and frequent alcohol use increase the risk of IPV, but the relationship between alcohol use and IPV (including recent and lifetime IPV victimization and perpetration) has not been well described among persons living with HIV (PWH) in sub-Saharan Africa.

**Methods:**

We used baseline data from the Drinker’s Intervention to Prevent Tuberculosis study. All participants were PWH co-infected with tuberculosis and had an Alcohol Use Disorders Identification Test – Consumption (AUDIT-C) positive score (hazardous drinking) and positive urine ethyl glucuronide test, indicating recent drinking. High-risk drinking was defined as AUDIT-C > 6 and/or alcohol biomarker phosphatidylethanol (PEth) ≥ 200 ng/mL. We measured IPV using the Conflict Tactics Scale. We estimated the association between alcohol use level and recent (prior six months) IPV victimization (recent perpetration was too low to study) using multivariable logistic regression models adjusted for gender, age, assets, education, spouse HIV status, religiosity, depressive symptoms, and social desirability. We additionally estimated the interaction of alcohol use and gender on IPV victimization and the association between alcohol use and lifetime victimization and perpetration.

**Results:**

One-third of the 408 participants were women. Recent IPV victimization was reported by 18.9% of women and 9.4% of men; perpetration was reported by 3.1% and 3.6% of women and men. One-fifth (21.6%) of those reporting recent IPV victimization also reported perpetration. In multivariable models, alcohol use level was not significantly associated with recent IPV victimization (p = 0.115), nor was the interaction between alcohol use and gender (p = 0.696). Women had 2.34 times greater odds of recent IPV victimization than men (p = 0.016). Increasing age was significantly associated with decreased odds of recent IPV victimization (p = 0.004).

**Conclusion:**

Prevalence of IPV victimization was comparable to estimates from a recent national survey, while perpetration among men was lower than expected. Alcohol use level was not associated with IPV victimization. It is possible that alcohol use in this sample was too high to detect differences in IPV. Our results suggest that women and younger PWH are priority populations for IPV prevention.

## Introduction

Intimate partner violence (IPV) and alcohol use are prevalent and interrelated public health issues [1]. Alcohol use has been causally linked to IPV perpetration [2] and identified as a risk factor for IPV victimization [3]. Alcohol use increases aggression in both men and women [4] and impairs one’s judgment, lessening capacity to negotiate non-violent conflict resolution (which in turn can lead to IPV) [5–7]. Evidence regarding the nature of the relationship between quantity and patterns of alcohol use and IPV is less clear. There is some evidence of a threshold effect with higher risk patterns of drinking (such as heavier and more frequent alcohol use) being associated with increased risk of perpetration as well as victimization [8, 9]. There is also some evidence of a linear relationship between alcohol use and IPV perpetration, suggesting a dose-response effect [8].

While both men and women can be perpetrators and victims of IPV, evidence suggests that violence is most frequently perpetrated by men towards women [10, 11] and the violence perpetrated by men is typically more severe and associated with greater injury [12, 13]. Unequal relationship power dynamics, prevailing social norms around gender and traditional constructs of masculinity that emphasize male exertion of power (at time through force) over females place women at greater risk of experiencing IPV [14, 15]. Unequal power dynamics and gendered economic systems may exacerbate vulnerability in relationships where younger women are reliant on older male partners for monetary support [16]. For these reasons, the majority of IPV research to date in sub-Saharan Africa has focused on perpetration among men and victimization among women. However, recent research globally has underscored the complexities of violence dynamics in intimate partnerships and bidirectional violence (also known as reciprocal violence where both partners perpetrate and experience IPV) is emerging as an understudied yet potentially key dynamic to consider when developing programming to reduce IPV [17, 18]. Understanding patterns of IPV has important implications for how and among whom interventions to reduce IPV should be implemented. For example, if violence were only perpetrated by men and experienced by women, intervention messaging would target men for behavior change and focus on provision of IPV resources for women only. However, if women also perpetrate and/or violence is largely bidirectional, intervention programming must target women as well or the couple and messaging must focus on behavior change among both partners.

Research exploring directionality of IPV beyond male perpetration and female victimization in sub-Saharan Africa has been limited but a recent study among persons living with HIV (PWH) in Malawi found that one quarter of all IPV (25.4%) experienced was bidirectional [19]. Recent data from a large community-based cohort study in central and western Uganda found high self-reported rates of lifetime IPV perpetration among women and lifetime IPV victimization among men, with the majority of persons experiencing IPV reporting both victimization and perpetration [20]. These findings suggest additional research is needed to better characterize dynamics of IPV in this setting.

Heavy alcohol use and HIV commonly co-occur in Uganda and together may have severe effects on IPV. Studies have identified alcohol use and IPV victimization as risk factors for incident and prevalent HIV infection among women in Uganda [21–23] and evidence from throughout sub-Saharan Africa suggests that women living with HIV experience IPV victimization at higher rates than women who are not living with HIV [24, 25]. However, comparable bodies of work exploring synergism between HIV, alcohol use and other patterns of IPV (such as perpetration among women, victimization among men and bidirectional violence) among PWH are lacking in this setting, underscoring critical gaps in our understanding of the relationship between these intersecting health issues.

To address this need, we sought to examine associations between alcohol use severity and directionality of physical IPV among a sample of PWH who are co-infected with TB and engage in heavy alcohol use in Uganda. We provide recent and lifetime estimates for physical IPV victimization and perpetration for men and women and explore gender differences in the association between severity of alcohol use and IPV. We hypothesized that higher risk drinking would be associated with all patterns of IPV and that gender differences would be observed (e.g., heavy alcohol use among men would be more strongly associated with IPV perpetration than heavy alcohol use among women).

## Materials and methods

### Study design and data collection

The Drinker’s Intervention to Prevent TB (DIPT) study (Clinical Trial number NCT03492216) is an ongoing randomized controlled trial being conducted in four communities in Southwestern Uganda among PWH wo are co-infected with TB and engage in heavy alcohol use. Study methods have previously been described in detail in the published study protocol [26]. In brief, DIPT uses a 2 × 2 factorial design; eligible participants were recruited from healthcare clinics and enrolled participants were randomly allocated to one of four study arms: (1) control (2) financial incentive contingent on reduced alcohol use (3) financial incentive contingent on high isoniazid (INH) adherence and (4) financial incentive contingent on both reduced alcohol use and high INH adherence. Participants across study arms initiated a 6-month course of INH. Participant eligibility criteria included being a PWH, having a positive AUDIT-C score (≥ 3 for women and ≥ 4 for men, the recommended cutoff for hazardous alcohol use) and having a positive urine ethyl glucuronide test (an objective measure indicative of recent alcohol use, using a commercial dipstick with a cutoff off of 300 ng/mL). Additional inclusion criteria and ineligibility criteria are described in the published study protocol [26].

Once a participant was enrolled in the study, they completed a baseline assessment which included a 45-minute interviewer-administered survey and a blood draw. Survey topics included sociodemographic variables, measures of mental and physical health status, self-reported ART adherence and alcohol use. Blood samples were tested for phosphatidylethanol (PEth), viral load, and CD4 count. PEth was extracted from dried blood spots and levels measured using LC/ MS-MS for the 16:0/18:1 homologue [27]. HIV viral load was measured using a Cepheid Xpert HIV-1 RNA assay run on an existing GeneXpert platform in Mbarara, Uganda. Participants were then randomized to one of the four study arms using methods previously described and followed up for 12 months [26]. Baseline data collection occurred between May 2018 and August 2021 (n = 680); the analytic sample was restricted to participants who completed their baseline visit after the IPV questions were added to the interviews in August 2019.

### Variables

Our primary dependent variable of interest was recent experiences of physical IPV. Our secondary dependent variable of interest was lifetime experiences of physical IPV. Both variables were measured using an adapted version of the conflict tactics scale (CTS), a globally validated measure of IPV [28]. Participants were asked about both IPV perpetration and victimization, with recall periods of (1) ever in their lifetime and (2) recently within the past 6 months. To measure lifetime IPV victimization, participants were asked, “Have any of your sexual partners ever done any of the following: Pushed, pulled, slapped, or held you down? Punched you? Kicked you or dragged you? Tried to strangle or burn you? Threatened or attacked you with a gun/knife/other weapon?”. Participants who replied yes to lifetime IPV victimization were then asked whether this had occurred in the prior 6 months (*recent* IPV victimization). To measure lifetime IPV perpetration, participants were asked “Have you ever physically hurt or threatened a sexual partner, including: Pushed, pulled, slapped, or held him/her down? Punched him/her? Kicked or dragged him/her? Tried to strangle or burn him/her? Threatened or attacked him/her with a gun/knife/other weapon?” Participants who replied yes to lifetime IPV perpetration were then asked whether this had occurred in the prior 6 months (*recent* IPV perpetration).

From these questions, we created IPV variables with the following four categories for both recall periods: no IPV, perpetration only, victimization only, and both perpetration and victimization. However, due to a small number of participants reporting only recent IPV perpetration, we were unable to use this variable for multivariable analysis and chose to focus our main analyses on recent experiences of IPV victimization (i.e., those who reported recent victimization regardless of if they also reported perpetration).

Our main independent variable of interest was severity of alcohol use, defined using both self-report and PEth. Participants were considered positive for the heaviest category of alcohol use if they self-reported an Alcohol Use Disorders Identification Test – Consumption (AUDIT-C [29, 30], modified to reflect prior 3 months use) score > 6 and/or had PEth results ≥ 200 ng/mL. Given that a positive AUDIT-C (using the validated cut-off of ≥ 3 for women, ≥ 4 for men) and positive ETG were eligibility criteria for participation in the study, we used high cutoffs for PEth and AUDIT-C (based on previous work) to differentiate between heavy alcohol use and the heaviest level of alcohol use. There is some evidence in the existing literature that measures capturing additional domains of alcohol use (such as the full 10-item AUDIT) are more strongly correlated with IPV than measures of consumption (captured in the AUDIT-C) [31]. To explore this, we undertook an additional exploratory analysis, using a combined alcohol measure of PEth (≥ 200 ng/mL) and prior year AUDIT scores (using a cutoff of AUDIT ≥ 11 for men and ≥ 9 for women [32, 33]) to differentiate between levels of alcohol use.

Demographic covariates included participant gender, age, and education (dichotomized as more than a primary education). Spouse HIV status was categorized as unknown, HIV-negative, HIV-positive, or not married (no spouse). A household asset index was created based on durable goods, housing quality and energy sources, using principal components analysis [34]. The participants were categorized as low (bottom 40%), middle (middle 40%), and high (top 20%). We used the Duke University Religion Index (DUREL) to measure participants’ intrinsic religiosity (subscale 3) [35], and the Center for Epidemiological Studies – Depression (CES-D) scale to assess depression [36]. A score of ≥ 16 on the CES-D was used to identify those with symptoms of depression. The 28-item Marlowe-Crowne Social Desirability Scale (SDS) was used to measure social desirability as a continuous scale [37].

### Statistical analyses

We calculated frequencies,medians and interquartile ranges (IQR), overall and by participant gender. We reported differences in sociodemographic and behavioral variables by gender. We examined associations with recent IPV victimization using unadjusted and adjusted logistic regression models. The multivariable model included the following variables, chosen a priori: heavy alcohol use, participant gender, age, education, spouse HIV status, household asset index, intrinsic religiosity, symptoms of depression and social desirability score. We also examined whether there was an interaction between alcohol use and participant gender in the main multivariable model. Several exploratory analyses were also performed. We assessed whether there was an interaction between participant gender and age in the main multivariable model. Recognizing that prior IPV perpetration and victimization are risk factors for subsequent violence and individuals may be less likely to report IPV (especially perpetration) in their current relationship due to social desirability, we also explored associations with lifetime victimization and lifetime perpetration. Again, small cell sizes for “perpetration only” precluded our ability to explore associations by directionality of lifetime IPV. Finally, we explored associations with recent IPV victimization using a second combined alcohol use measure comprised of full 10-item AUDIT score and PEth level. We also performed a post-hoc analysis to examine whether personal income, measured by daily wages, was also associated with the outcome. We considered a p-value of 0.10 as significant when assessing interactions and a p-value of 0.05 as significant when assessing main effects.

Internal consistency for the three scale measures (CES-D, DUREL and SDS) was assessed using Cronbach’s alpha coefficient for which a score equal or higher than 0.7 is acceptable [38]. Cronbach’s alpha coefficients for the CES-D, SDS and DUREL were 0.88, 0.79 and 0.90, respectively, suggesting good internal consistency.

### Ethical considerations

Study enrollment procedures including the informed consent process occur in a private one on one setting to ensure participant confidentiality. Written informed consent is obtained at two stages: prior to the screening process and again after eligibility has been confirmed. Informed consent documents are provided in both English or Runyankole depending on the participant’s preference. Participants are informed of their right to enroll or not enroll and are provided with a list of potential risks associated with the study including loss of confidentiality. To ensure anonymity, participants are also informed that any published findings will be deidentified and that only members of the study team will have access to their personal information. This study was approved by the Institutional Review Board at University of California, San Francisco; the Mbarara University of Science and Technology Research Ethics Committee; the Makerere University School of Medicine Research Ethics Committee; and the Ugandan National Council for Science and Technology.

## Results

### Sociodemographic and behavioral characteristics, prevalence of IPV and heavy alcohol use among DIPT participants

The analytic sample included data from baseline visits of 408 study participants. One hundred and thirty-two participants (32%) were female and median participant age was 39 years [IQR 32–46 years]. Two hundred and thirty-one participants (57%) were currently married and 150 (37%) had a spouse that was also living with HIV. Most participants (n = 332, 81%) did not have more than a primary school education. Additional sociodemographic and behavioral characteristics from baseline visits can be found in Table1.

**Table 1:** Baseline characteristics of DIPT Study participants, overall and stratified by sex

	Overall (n = 408)	Female (n = 132)	Male (n = 276)
	**N (%)**	**N (%)**	**N (%)**
**Demographics**			
Age (median [IQR])	39 [32–46]	38 [30–46]	40 [33–46]
Level of Education			
Primary and below	332 (81.4)	121 (91.7)	211 (76.5)
Above primary education	76 (18.6)	11 (8.3)	65 (23.6)
Household Asset Index			
Low	185 (45.5)	67 (51.2)	118 (42.8)
Middle	147 (36.1)	40 (30.5)	107 (38.8)
High	75 (18.4)	24 (18.3)	51 (18.5)
DUREL – intrinsic religiosity (median [IQR])	15 [12–15]	15 [12–15]	15 [12–15]
CESD			
No depressive symptomology (< 16)	368 (90.2)	121 (91.7)	247 (89.5)
Depressive symptomology (≥ 16)	40 (9.8)	11 (8.3)	29 (10.5)
Social Desirability Score (median [IQR])	20 [18–23]	21 [18–23]	20 [18–22]
Marital status			
Married, living together	201 (49.3)	49 (37.1)	152 (55.1)
Married, not living together	30 (7.4)	9 (6.8)	21 (7.6)
Divorced/separated	105 (25.7)	37 (28.0)	68 (24.6)
Widowed	31 (7.6)	22 (16.7)	9 (3.3)
Never married and not living together	41 (10.1)	15 (11.4)	26 (9.4)
Spouse HIV serostatus			
Unknown	11 (2.7)	7 (5.3)	4 (1.5)
Negative	70 (17.2)	17 (12.9)	53 (19.2)
Positive	150 (36.8)	34 (25.8)	116 (42.0)
Not married (no spouse)	177 (43.4)	74 (56.1)	103 (37.3)
**Alcohol use**			
AUDIT-C (median [IQR])	5.5 [4–8]	5 [4–7]	6 [4–8]
AUDIT-C			
≤ 6	254 (62.3)	93 (70.5)	161 (58.3)
> 6	154 (37.8)	39 (29.6)	115 (41.7)
AUDIT (median [IQR])	11 [7–18]	8 [5–14]	13 [8–19]
AUDIT			
< 9 (women); <11 (men)	170 (42.3)	67 (51.2)	103 (38.0)
≥ 9 (women); ≥11 (men)	232 (57.7)	64 (48.9)	168 (62.0)
PEth level			
< 200	170 (41.7)	89 (67.4)	81 (29.4)
≥ 200	238 (58.3)	43 (32.6)	195 (70.7)
Alcohol use: PEth ≥ 200 and/or AUDIT-C > 6?			
No	124 (30.4)	67 (50.8)	57 (20.7)
Yes	284 (69.6)	65 (49.2)	219 (79.4)
Alcohol use: PEth ≥ 200 and/or AUDIT ≥ 9 (women); ≥11 (men)			
No	89 (21.9)	49 (37.1)	40 (14.6)
Yes	317 (78.1)	83 (62.9)	234 (85.4)
**IPV**			
Lifetime violence perpetration			
No	347 (85.1)	119 (90.2)	228 (82.6)
Yes	60 (14.7)	12 (9.1)	48 (17.4)
Don’t know	1 (0.3)	1 (0.8)	0 (0.0)
Recent violence perpetration (past 6m)			
No	393 (96.6)	127 (97.0)	266 (96.4)
Yes	14 (3.4)	4 (3.1)	10 (3.6)
Lifetime violence victimization			
No	293 (71.8)	82 (62.1)	211 (76.5)
Yes	115 (28.2)	50 (37.9)	65 (23.6)
Recent violence victimization (past 6m)			
No	357 (87.5)	107 (81.1)	250 (90.6)
Yes	51 (12.5)	25 (18.9)	26 (9.4)
Directional lifetime IPV			
Yes, perpetration only	27 (6.6)	2 (1.5)	25 (9.1)
Yes, victimization only	82 (20.2)	40 (30.5)	42 (15.2)
Both victimization and perpetration	33 (8.1)	10 (7.6)	23 (8.3)
None	265 (65.1)	79 (60.3)	186 (67.4)
Directional recent IPV (past 6m)			
Yes, perpetration only	3 (0.7)	2 (1.5)	1 (0.4)
Yes, victimization only	40 (9.8)	23 (17.6)	17 (6.2)
Both victimization and perpetration	11 (2.7)	2 (1.5)	9 (3.3)
None	353 (86.7)	104 (79.4)	249 (90.2)
Any recent IPV (past 6m)			
No	353 (86.7)	104 (79.4)	249 (90.2)
Yes	54 (13.3)	27 (20.6)	27 (9.8)

Using the AUDIT-C/PEth combined alcohol use measure, 284 participants (70%) fell into the heaviest alcohol use category (PEth ≥ 200 and/or AUDIT-C > 6). Using the AUDIT/PEth combined alcohol use measure, 317 participants (78%) fell into the heaviest alcohol use category (PEth ≥ 200 and/or AUDIT ≥ 9 (women) or ≥ 11 (men)). Recent and lifetime IPV victimization were more prevalent than IPV perpetration. Recent IPV victimization was reported by 51 participants (13%) while lifetime IPV victimization was reported by 115 participants (28%). Recent IPV perpetration was reported by 14 (3%) while lifetime perpetration was reported by 60 participants (15%). Bidirectional violence accounted for 20% of recent IPV among the 54 participants reporting any recent IPV, and 23% of lifetime IPV among the 142 participants reporting any lifetime IPV (Table1).

Mean age, education level, marital status, spouse HIV status, level of alcohol use and all IPV variables except for recent IPV perpetration significantly differed by gender. Female participants were generally younger than males (median age: 38 years vs. 40 years), and a greater proportion of female than males had completed schooling (8% vs. 24% had greater than primary education), were unmarried (44% vs. 63%), did not have a spouse living with HIV (26% vs. 42%), did not fall into the heaviest alcohol use category (PEth ≥ 200 and/or AUDIT-C > 6) (49% vs. 79%) and did not report lifetime IPV perpetration (9% vs. 17%). A greater proportion of females than males reported both recent (19% vs. 9%) and lifetime (38% vs. 24%) IPV victimization. A smaller proportion of females than males reported recent (2% vs. 3%) bidirectional IPV (Table1).

### Unadjusted association between sociodemographic and behavioral characteristics and recent IPV victimization

Table2 provides the bivariate associations and unadjusted odds ratios for any recent IPV victimization among DIPT participants; we did not proceed with analyses of recent perpetration given the small numbers. Education level, household asset index, DUREL, CES-D, SDS and spouse HIV status were not associated with recent IPV victimization. Women had 2.25 times greater odds of recent IPV victimization than men (OR 2.25, 95% CI 1.24, 4.07, p = 0.008). Age was also significantly associated with IPV; odds of recent IPV decreased as age increased (OR 0.58 per 10 years, 95% CI 0.42–0.81 p = 0.001). Alcohol use level was not associated with experiences of recent IPV victimization in unadjusted analysis (Odds Ratio (OR) 1.32, 95% Confidence Interval (CI) 0.68, 2.57, p = 0.417 using the PEth/AUDIT-C measure and OR 1.17, 95% CI 0.56, 2.45, p = 0.670 using the exploratory PEth/AUDIT measure). Our interaction term between alcohol use level and participant sex was also not significant (p = 0.786).

**Table 2:** Bivariate associations and unadjusted Odds Ratios (OR) and 95% Confidence Intervals (CI) for any recent IPV victimization among DIPT Study participants (n = 408)

	Any recent IPV victimization?			
	**No (n = 357)** **N (%)**	**Yes (n = 51)** **N (%)**	**Unadjusted** **OR (95% CI)**	**p-value**
Alcohol use: PEth ≥ 200 and/or AUDIT-C > 6?				0.417
No	111 (89.5)	13 (10.5)	1.00	
Yes	246 (86.6)	38 (13.4)	1.32 (0.68, 2.57)	
Alcohol use: PEth ≥ 200 and/or AUDIT ≥ 9 (women); ≥11 (men)?				0.670
No	79 (88.8)	10 (11.2)	1.00	
Yes	276 (87.1)	41 (12.9)	1.17 (0.56, 2.45)	
Gender				0.008
Female	107 (81.1)	25 (18.9)	2.25 (1.24, 4.07)	
Male	250 (90.6)	26 (9.4)	1.00	
Alcohol use** X Gender				Intxn p = 0.786
Female: not highest alcohol	57 (85.1)	10 (14.9)	3.16 (0.82, 12.09)	0.093
Female: highest alcohol	50 (76.9)	15 (23.1)	5.40 (1.47, 19.77)	0.011
Male: not highest alcohol	54 (94.7)	3 (5.3)	1.00	
Male: highest alcohol	196 (89.5)	23 (10.5)	2.11 (0.61, 7.30)	0.237
Age (median [IQR])	40 [32–47]	35 [29–42]		
Age (per 10 years)			0.58 (0.42, 0.81)	0.001
More than a primary education				0.339
No	288 (86.8)	44 (13.3)	1.51 (0.65, 3.49)	
Yes	69 (90.8)	7 (9.2)	1.00	
Household Asset Index				0.646
Low	160 (86.5)	25 (13.5)	1.52 (0.63, 3.68)	
Middle	128 (87.1)	19 (12.9)	1.44 (0.58, 3.60)	
High	68 (90.7)	7 (9.3)	1.00	
DUREL – intrinsic religiosity (median [IQR])	15 [12–15]	15 [12–15]		
DUREL – intrinsic religiosity (per 1 point)			1.03 (0.94, 1.12)	0.525
CESD				1.000
No depressive symptomology (< 16)	322 (87.5)	46 (12.5)	1.00	
Depressive symptomology (≥ 16)	35 (87.5)	5 (12.5)	1.00 (0.37, 2.68)	
Social Desirability Score (median [IQR])	20 [18–22]	20 [16–23]		
Social Desirability Score (per 1 point)			0.96 (0.89, 1.05)	0.385
Spouse HIV serostatus				0.361
Unknown	8 (72.7)	3 (27.3)	2.54 (0.57, 11.39)	
Negative	61 (87.1)	9 (12.9)	1.00	
Positive	129 (86.0)	21 (14.0)	1.10 (0.48, 2.55)	
Not married	159 (89.8)	18 (10.2)	0.77 (0.33, 1.80)	

**Alcohol use: highest = PEth ≥ 200 and/or AUDIT-C > 6; not highest = PEth < 200 and AUDIT-C ≤ 6

### Adjusted association between alcohol use category and recent IPV victimization

In the adjusted model 1 (See Table3), using the PEth/AUDIT-C combined measure, the adjusted odds ratio for alcohol use level and IPV was 1.81 (95% CI 0.87, 3.80), but the relationship was not statistically significant (p = 0.115). In the adjusted model that further included an interaction term between alcohol use level and gender, the interaction term was not significant (p = 0.696). In an adjusted model including an interaction term between age and gender, the interaction term was also not significant (p = 0.152). In an adjusted model using the exploratory PEth/AUDIT combined alcohol measure, the odds ratio for alcohol use level and IPV was 1.61 (95% CI 0.71, 3.62) but the relationship was not statistically significant (p = 0.250). In further sensitivity analyses, personal income was not associated with recent IPV victimization and did not impact the association between alcohol use and recent IPV victimization (data not shown).

**Table 3:** Adjusted Odds Ratios (OR) and 95% Confidence Intervals (CI) for any recent IPV victimization among DIPT Study participants (n = 405)

	Model 1		Model 2		Model 3 (exploratory)		Model 4 (exploratory)	
	**Adjusted OR** **(95% CI)**	**p-value**	**Adjusted OR** **(95% CI)**	**p-value**	**Adjusted OR** **(95% CI)**	**p-value**	**Adjusted OR** **(95% CI)**	**p-value**
Alcohol use: PEth ≥ 200 and/or AUDIT-C > 6?		0.115				0.129		
No	1.00		-		1.00		-	
Yes	1.81 (0.87, 3.80)		-		1.79 (0.85, 3.78)		-	
Alcohol use: PEth ≥ 200 and/or AUDIT ≥ 9 (women); ≥11 (men)?								0.250
No	-		-		-		1.00	
Yes	-		-		-		1.61 (0.71, 3.62)	
Gender		0.016						0.024
Female	2.34 (1.17, 4.67)		-		-		2.19 (1.11, 4.34)	
Male	1.00		-		-		1.00	
Alcohol use* X Gender				Intxn p = 0.696				
Female: not highest alcohol	-		2.96 (0.74, 11.77)	0.124	-		-	
Female: highest alcohol	-		4.78 (1.24, 18.46)	0.023	-		-	
Male: not highest alcohol	-		1.00		-		-	
Male: highest alcohol	-		2.21 (0.63, 7.75)	0.217	-		-	
Age (per 10 years)	0.59 (0.41, 0.84)	0.004	0.59 (0.41, 0.84)	0.004	-		0.59 (0.41, 0.84)	0.004
Age x Gender						Intxn p = 0.152		
Female, per 10 years	-		-		0.45 (0.26, 0.77)	0.004	-	
Male, per 10 years	-		-		0.75 (0.47, 1.21)	0.238	-	
More than a primary education		0.520		0.517		0.552		0.515
No	1.34 (0.55, 3.30)		1.35 (0.55, 3.31)		1.31 (0.54, 3.22)		1.35 (0.55, 3.31)	
Yes	1.00		1.00		1.00		1.00	
Household Asset Index		0.546		0.540		0.504		0.565
Low	1.63 (0.63, 4.23)		1.64 (0.63, 4.26)		1.68 (0.65, 4.36)		1.62 (0.62, 4.22)	
Middle	1.23 (0.48, 3.19)		1.24 (0.48, 3.21)		1.23 (0.47, 3.20)		1.24 (0.48, 3.23)	
High	1.00		1.00		1.00		1.00	
DUREL – intrinsic religiosity (per 1 point)	1.03 (0.94, 1.14)	0.483	1.03 (0.94, 1.14)	0.481	1.03 (0.94, 1.13)	0.508	1.04 (0.94, 1.14)	0.443
CESD		0.806		0.820		0.719		0.861
No depressive symptomology (< 16)	1.00		1.00		1.00		1.00	
Depressive symptomology (≥ 16)	1.14 (0.40, 3.21)		1.13 (0.40, 3.19)		1.21 (0.43, 3.45)		1.10 (0.39, 3.08)	
Social Desirability Score (per 1 point)	0.96 (0.88, 1.05)	0.407	0.96 (0.88, 1.05)	0.401	0.96 (0.88, 1.05)	0.409	0.96 (0.87, 1.05)	0.330
Spouse HIV serostatus		0.111		0.112		0.113		0.098
Unknown	2.37 (0.45, 12.42)		2.33 (0.45, 12.16)		2.56 (0.48, 13.80)		2.44 (0.47, 12.75)	
Negative	1.00		1.00		1.00		1.00	
Positive	1.12 (0.47, 2.68)		1.12 (0.22, 1.33)		1.15 (0.48, 2.77)		1.09 (0.46, 2.61)	
Not married (no spouse)	0.54 (0.22, 1.33)		0.54 (0.22, 1.33)		0.56 (0.23, 1.38)		0.53 (0.21, 1.29)	

*Alcohol use: highest = PEth ≥ 200 and/or AUDIT-C > 6; not highest = PEth < 200 and AUDIT-C ≤ 6

### Association between socio-demographic and behavioral characteristics, alcohol use category and lifetime IPV victimization and perpetration among DIPT participants

Table4 provides the bivariate associations and adjusted odds ratios for lifetime IPV victimization and lifetime IPV perpetration among DIPT participants. Using the PEth/AUDIT-C combined measure, the adjusted odds ratio for alcohol use level and lifetime IPV victimization was not statistically significant (aOR 1.25, 95% CI 0.74, 2.09, p = 0.403). Education level, household asset index, DUREL, CES-D, SDS and spouse HIV status were also not associated with lifetime IPV victimization. Women had 2.10 times greater odds of lifetime IPV victimization than men (aOR 2.10, 95% CI 1.25, 3.50, p = 0.005). Using the PEth/AUDIT-C combined measure, the odds ratio for alcohol use level and lifetime IPV perpetration was in the expected direction (greater odds of IPV perpetration among those in the highest risk category) but the relationship was not statistically significant (aOR 1.89, 95% CI 0.88, 4.02, p = 0.100). Gender, education level, household asset index, DUREL, CES-D and spouse HIV status were also not associated with lifetime IPV perpetration. SDS was significantly associated with lifetime IPV perpetration, with decreased odds of reporting IPV perpetration as SDS score increased (aOR 0.91 per 1 point, 95% CI 0.83–0.99 p = 0.025).

**Table 4:** Bivariate associations and adjusted Odds Ratios (OR) and 95% Confidence Intervals (CI) for lifetime IPV among DIPT study participants. (exploratory)

	Lifetime IPV victimization				Lifetime IPV perpetration			
	**No (n = 293)** **N (%)**	**Yes (n = 115)** **N (%)**	Adjusted OR* **(95% CI)**	**p-value**	**No (n = 347)** **N (%)**	**Yes (n = 60)** **N (%)**	Adjusted OR** **(95% CI)**	**p-value**
Alcohol use: PEth ≥ 200 and/or AUDIT-C > 6?				0.403				0.100
No	89 (71.8)	35 (28.2)	1.00		113 (91.9)	10 (8.1)	1.00	
Yes	204 (71.8)	80 (28.2)	1.25 (0.74, 2.09)		234 (82.4)	50 (17.6)	1.89 (0.88, 4.02)	
Gender				0.005				0.252
Female	82 (62.1)	50 (37.9)	2.10 (1.25, 3.50)		119 (90.8)	12 (9.2)	0.65 (0.31, 1.36)	
Male	211 (76.5)	65 (23.6)	1.00		228 (82.6)	48 (17.4)	1.00	
Age (median [IQR])	40 [32–47]	38 [31–44]			39 [32–46]	39.5 [35-48.5]		
Age (per 10 years)			0.81 (0.64, 1.01)	0.063			1.14 (0.86, 1.52)	0.355
More than a primary education				0.506				0.894
No	236 (71.1)	96 (28.9)	1.23 (0.67, 2.28)		283 (85.5)	48 (14.5)	1.05 (0.50, 2.21)	
Yes	57 (75.0)	19 (25.0)	1.00		64 (84.2)	12 (15.8)	1.00	
Household Asset Index				0.268				0.895
Low	134 (72.4)	51 (27.6)	0.73 (0.39, 1.38)		158 (85.9)	26 (14.1)	0.84 (0.37, 1.91)	
Middle	109 (74.2)	38 (25.9)	0.59 (0.31, 1.12)		125 (85.0)	22 (15.0)	0.83 (0.37,1.87)	
High	49 (65.3)	26 (34.7)	1.00		63 (84.0)	12 (16.0)	1.00	
DUREL – intrinsic religiosity (median [IQR])	15 [12–15]	15 [12–15]			15 [12–15]	15 [11–15]		
DUREL – intrinsic religiosity (per 1 point)			1.01 (0.95, 1.08)	0.767			0.96 (0.89, 1.04)	0.311
CESD				0.516				0.089
No depressive symptomology (< 16)	266 (72.3)	102 (27.7)	1.00		318 (86.7)	49 (13.4)	1.00	
Depressive symptomology (≥ 16)	27 (67.5)	13 (32.5)	1.28 (0.61, 2.66)		29 (72.5)	11 (27.5)	2.06 (0.90, 4.70)	
Social Desirability Score (median [IQR])	20.2 [18-22.6]	20 [17–23]			20 [18–23]	19.5 [15.5–22]		
Social Desirability Score (per 1 point)			0.94 (0.88, 1.01)	0.079			0.91 (0.83, 0.99)	0.025
Spouse HIV serostatus				0.359				0.682
Unknown	7 (63.6)	4 (36.4)	1.15 (0.28, 4.81)		9 (81.8)	2 (18.2)	0.70 (0.08, 6.37)	
Negative	48 (68.6)	22 (31.4)	1.00		57 (81.4)	13 (18.6)	1.00	
Positive	108 (72.0)	42 (28.0)	0.84 (0.44, 1.58)		127 (85.2)	22 (14.8)	0.73 (0.34, 1.60)	
Not married	130 (73.5)	47 (26.6)	0.60 (0.32, 1.14)		154 (87.0)	23 (13.0)	0.61 (0.28, 1.35)	

* n = 405, ** n = 404

## Discussion

Recent IPV victimization was reported by 18.9% of women and 9.4% of men in our sample; recent perpetration was reported by 3.1% and 3.6% of women and men, respectively. Our findings were largely consistent with those found in the 2016 Uganda Demographic and Health Survey [39]. Our prevalence of recent victimization among women was also consistent with a meta-analysis among women living with HIV in sub-Saharan Africa which found a pooled prevalence of 18% for physical IPV [40]. Our findings around perpetration diverged from DHS findings; we observed gender symmetry in recent IPV perpetration (3.1% percent of women and 3.6% of men), while our estimates were consistent with DHS estimates for perpetration among women the DHS estimates among men were much higher (9%) [39]. This divergent finding may be due to true differences in our sample of PWH who engage in heavy alcohol use relative to the DHS sample but are more likely a product of underreporting (especially the low rates among men who are more likely to underreport perpetration [41]).

Reported rates of perpetration in our sample were so low, in fact, that it precluded our ability to look at associations between directionality of IPV and severity of alcohol use. However, we were still able to explore gendered differences in the association between experiences of recent IPV victimization and level of alcohol use. We hypothesized that persons in the heaviest alcohol use category would have greater odds of experiencing IPV victimization, and we observed higher levels of IPV in the high-risk group, but the associations between alcohol use and IPV victimization and perpetration were not statistically significant. However, sociodemographic factors such as being female and younger were associated with increased risk of IPV victimization. We also explored lifetime victimization and perpetration and found an association between lifetime IPV perpetration and SDS score (persons with lower scores were more likely to report perpetration), suggesting estimates of IPV perpetration in this sample (and by extension, bidirectional IPV) are likely underestimated due to underreporting. These findings have important implications for public health and future research as well as intervention development and our recommendations are described below. Namely, they underscore that women and young person’s remain priority populations for IPV programming and services. They also highlight the challenge of quantifying the true public health burden of IPV and accurate identification of perpetrators for targeted intervention.

A challenge to conducting IPV research globally is the subjective nature of self-reported measures and the absence of an objective alternative. Self-report of experiences of IPV are subject to bias due to recall issues as well as social desirability [42]. Discrepancies between rates of victimization and perpetration (as we observed in our study) frequently occur regardless of gender, with victimization reported at much higher rates than perpetration [43–46]. Underreporting of IPV perpetration is frequently attributed to social desirability bias [47, 48]. Prior work suggests that social desirability is correlated with both victimization and perpetration of IPV but perpetration is more susceptible to social desirability and this is suggested by our findings [42]. In future work, use of real-time data collection approaches that reduce risk of recall bias for (such as a daily diary [49]) and data collection methods that improve participant privacy (use of self-administered audio-assisted computer interviews (ACASI) instead of interviewer administered questions) may improve the accuracy of IPV responses [43]. Mixed methods work to clarify the severity and frequency of IPV and identify acts of IPV that may not be reported (i.e., culturally understood to constitute violence) is also needed.

The relationship between alcohol use and IPV victimization and perpetration is well established, and we expected being in the heaviest alcohol use category to be associated with both victimization and perpetration. The lack of significant association between level of alcohol use and IPV in our sample may be a product of our eligibility criteria. By design, all participants in the present study had AUDIT-C scores indicative of hazardous drinking, and EtG evidence of recent alcohol consumption; two thirds had PEth levels indicating excessive drinking or more. The level of alcohol use in our sample may have been too high to detect a threshold between alcohol use and IPV. In addition, our lack of a true reference group of low-level users/abstainers may have also reduced our ability to detect a dose response effect; prior studies reporting dose response effects typically included reference groups with no/low levels of alcohol use [50]. Although not statistically significant, the strength of the association between alcohol use level and recent IPV victimization increased in our adjusted models (from an aOR of 1.32 to an aOR of 1.81) which is more consistent with prior findings than the unadjusted results. The association between alcohol use level and lifetime perpetration approached significance and misclassification of individuals due to underreporting (i.e., perpetrators reporting no violence) may have biased this estimate towards the null.

While our findings suggest that IPV victimization is prevalent among both men and women living with HIV, it is important to note that women who experience IPV are more likely to sustain injuries from that violence [39], underscoring gendered differences in the public health burden associated with IPV. Use of validated IPV measures that capture the frequency and severity of violence experienced (as recommended above) will better characterize the public health burden attributable to violence in this population. However, understanding that women perpetrate IPV in this setting and that a sizable minority of participants experience bidirectional IPV is critical information for public health planning and highlights the need to focus IPV prevention messaging around the couple (as opposed to exclusively targeting men).

As with all research, this analysis had limitations. The data are cross-sectional, precluding our ability to infer directionality or temporality of observed associations. We also lacked a true reference group for our exposure of interest (alcohol use) which may have affected our ability to detect associations of interest. Balanced recruitment by gender was not part of the DIPT study design. Participants were identified based on self-report of alcohol use during HIV care visits and 2/3 of the sample is comprised of male participants, which reflects the higher rates of alcohol use among men in Uganda. We included an interaction term with gender and alcohol use level to explore the role of gender in the relationship between alcohol use and IPV. Had this interaction term been significant we would have stratified the analysis based on effect modification on the multiplicative scale and reported gender models separately. Finally, our IPV outcomes were self-reported and subject to bias. However, this analysis also had strengths. We utilized an alcohol biomarker to supplement self-report, making our estimates more accurate than solely subjective alcohol use measures. In addition, we explored IPV directionality, an under-researched topic in sub-Saharan Africa. Finally, our analysis identified populations to target for intervention development. Women in our sample were at increased odds of experiencing physical IPV, a finding consistent with a large body of global research. Age was also inversely associated with IPV victimization suggesting young adults should be targeted for intervention programming. Adolescent women and girls are at greater risk of experiencing gender-based violence and as such, are a widely recognized priority population for violence prevention programming [51]. Our interaction term between age and gender was in the expected direction but not statistically significant, suggesting that both men and women of younger age in our sample are at increased risk of experiencing IPV and would benefit from IPV prevention programming. School-based intervention programming offers the opportunity to change norms around violence perpetration towards intimate partners at an early age. Evidence from South Africa suggest that school-based interventions to address HIV and IPV can reduce experiences of IPV victimization among both boys and girls [52]. Evidence-based couples’ interventions that seek to change gender norms that promote violence could also be adapted to address bidirectional violence and victimization among men [53–55]. Such interventions could be integrated into HIV care service delivery, reducing the resources required to implement them, as recommended by Liverpool VCT Care and Treatment, Sexual Violence Research Initiative and World Health Organization in their report on strengthening the response to gender-based violence and HIV in sub-Saharan Africa [56].

## Conclusion

IPV is a pervasive public health issue in Uganda and recent IPV was common among our sample of PWH who are co-infected with TB and engage in heavy alcohol use. Steps must be taken to improve measurement of self-reported IPV perpetration such as use of self-administered questionnaires. While women are more likely to experience IPV, programs to prevent IPV in Uganda should focus on addressing IPV at a young age when risk of victimization is higher and target both men and women. Extant literature suggests integration of such interventions into schools (i.e., classroom delivery) and HIV services (i.e., couples-based delivery) offers opportunities for low resource intervention delivery.

## Data Availability

Individuals interested in accessing the data should contact Dr. Judy Hahn. (Judy.Hahn@ucsf.edu).
